# Correlating bacterial shedding with fecal corticosterone levels and serological responses from layer hens experimentally infected with *Salmonella* Typhimurium

**DOI:** 10.1186/s13567-017-0414-9

**Published:** 2017-02-06

**Authors:** Pardeep Sharma, Vivek V. Pande, Talia S. Moyle, Andrea R. McWhorter, Kapil K. Chousalkar

**Affiliations:** School of Animal and Veterinary Sciences, The University of Adelaide, Roseworthy, SA 5173 Australia

## Abstract

*Salmonella* Enteriditis and *Salmonella* Typhimurium are commonly isolated during egg-related outbreaks of salmonellosis and represent a significant international public health issue. In Australia, *Salmonella* Typhimurium is the most common serovar identified in egg product related foodborne outbreaks. While a number of studies have investigated *Salmonella* shedding and host responses to infection, they have been conducted over a short time period. The present study sought to characterise bacterial shedding and host responses to infection in hens infected with only *Salmonella* Typhimurium or co-infected with both *Salmonella* Typhimurium and *Salmonella* Mbandaka over a 16 week period. *Salmonella* shedding was quantified using the most probable number and qPCR methods and was highly variable over the course of the experiment. On day 1, fecal corticosterone metabolites in birds infected with *Salmonella* Typhimurium (674.2 ± 109.3 pg/mg) were significantly higher than control (238.0 ± 12.62 pg/mg) or co-infected (175.4 ± 8.58 pg/mg) birds. The onset of lay occurred between weeks 6–8 post-infection (pi) and Fecal corticosterone metabolite (FCM) concentrations increased in both control and co-infected birds. Antibody responses to infection were monitored in both serum and yolk samples. *Salmonella* Typhimurium specific antibody was lower in co-infected animals than monoinfected animals. Bacterial loads in internal organs were characterised to determine persistence. Spleen, liver and caecal tonsils were positive for bacteria in both groups, indicating that *Salmonella* was not cleared from the birds and internal organ colonization could serve as a reservoir for continued bacterial shedding.

## Introduction

Commercial poultry are often persistently infected with non-typhoidal serovars of *Salmonella enterica*. Eggs and raw egg based food products are often identified as the source of *Salmonella* during outbreaks of human gastrointestinal disease [1]. Thus, the zoonotic potential of *Salmonella* represents a significant global public health concern. In North America and Europe, the most common serovar isolated during egg-related outbreaks is *Salmonella* Enteritidis followed by *Salmonella* Typhimurium [2]. Strains of *Salmonella* Typhimurium, however, are most frequently identified during Australian outbreaks of egg-related cases of salmonellosis [1].

Over the past several years, the incidence of human cases of salmonellosis in Australia has been increasing. In 2011, the total number of food related disease outbreaks had increased to over 150 and 38.4% were attributed to *Salmonella* [1]. Over the same period, the number of cases linked directly with eggs increased from 20.8 to 44.8% [1]. Despite improvements of on-farm control strategies, *Salmonella* Typhimurium remains a significant problem within the Australian layer industry [3].

Due to the public health importance of contaminated eggs, understanding the dynamics of *Salmonella* Typhimurium shedding patterns and associated host responses to infection is of critical importance. Previous experimental infection trials have examined egg contamination and internal organ colonization of layer hens. These studies, however, have infected birds at different ages, using a variety of inoculation methods [4–7] limiting the degree to which the data can be directly compared. Moreover, the data obtained from these investigations was collected for 3–4 weeks pi. The productive lifetime of a layer hen, however, can extend beyond 50 weeks of age and few studies have investigated extended bacterial shedding dynamics, egg contamination and host responses to infection.During productive lifespan, layer hens may also experience many physiological and environmental stressors, such as overcrowding, extreme temperature variation and the onset of lay that may lead to increased fecal shedding of *Salmonella* [8–11]. Stress has also been linked with impaired immunity [8, 9, 12, 13] which may increase intestinal colonization by enteric pathogens such as *Salmonella* [14]. The host immune response to *Salmonella* infection may also contribute to increased corticosterone levels however, relationship between persistent *Salmonella* colonisation and stress in birds is unclear.

In the Australian egg industry, *Salmonella* Typhimurium is frequently isolated from eggshell surfaces but it is not the only serovar isolated from egg farms [15, 16]. The poultry farm environment is often contaminated with multiple serovars [15–17]. Field epidemiological investigations suggested that *Salmonella* Mbandaka was commonly isolated along with *Salmonella* Typhimurium in layer flocks without any clinical signs in chickens [16, 18]. *Salmonella* Mbandaka has not been associated with any egg related outbreaks in Australia [19], although this serovar has been associated with egg product related *Salmonella* outbreaks in the US [20].

Competition between co-infecting strains may affect the dynamics of one or more serovars. Layer hens environmentally infected with *Salmonella* Kentucky, for example, mitigated *Salmonella* Enteritidis colonisation of internal organs [21]. In addition, coinfection of layer hens with *Salmonella* Enteritidis, *Salmonella* Gallinarum and *Salmonella* Isangi has recently been shown to enhance disease in infected birds [22]. To date, there have been limited studies investigating how co-infection affects the dynamics of *Salmonella* Typhimurium shedding as well as host responses to infection.

Our hypothesis was that *Salmonella* Mbandaka can affect the shedding of *Salmonella* Typhimurium and internal organ colonization. We have conducted a 16 week infection trial, using layer hens reared free from exogenous *Salmonella.* Results from a companion study demonstrated that over the 16 week infection period, bacterial shedding was variable and that vertical transmission of *Salmonella* Typhimurium DT9 into egg internal did not occur [23]. The aims of the present study were to correlate fecal shedding and egg contamination patterns with host responses to infection (single and mixed) including fecal corticosterone levels as a marker of the host stress response as well as levels of *Salmonella* Typhimurium specific antibodies in the serum and yolk. A final aim of this study was to characterise persistence of *Salmonella* infection in peripheral organs.

## Materials and methods

### Birds

Fertile eggs were obtained from a commercial brown layer flock hatchery. Eggs were fumigated using formaldehyde and incubated for 21 days. A total of 32 pullets were hatched and raised in floor pens in positive pressure rooms within an animal housing facility located on the Roseworthy Campus of the University of Adelaide. The rooms within this facility, all animal cages, trays, and feeders had previously been cleaned and decontaminated using FoamCleanS and SaniGuard (Chemtall, Australia). At 10 weeks of age, birds were divided into three treatment groups: control (*n* = 4), *Salmonella* Typhimurium (*n* = 14) and *Salmonella* Typhimurium + *Salmonella* Mbandaka (*n* = 14) and housed individually in cages in separate rooms. Fumigated feed and sanitised water (Aquatabs, Ireland) were provided ad libitum to all birds. Feed, water and fecal samples were screened for *Salmonella* fortnightly by culture method as described previously [16]. This experiment was performed according to the Australian Code for the Care and Use of Animals for Scientific Purposes and was approved by the University of Adelaide Animal Ethics Committee (approval number: S-2014-008).

### Bacterial isolates

Single isolates of *Salmonella* Typhimurium definitive type 9 (DT9) and *Salmonella* Mbandaka were used in this study. These *Salmonella* had been previously isolated from samples collected from layer hen farms during a previous epidemiology study [16] and serotyped at the *Salmonella* Reference Laboratory, Institute of Veterinary Medical Science (IMVS), Adelaide, South Australia.

### Challenge experiment

At 14 weeks of age, just prior to lay, hens were orally inoculated with 1 × 10^9^ colony forming units (CFU) of either *Salmonella* Typhimurium DT9 or a combination containing equal amounts of both *Salmonella* Typhimurium DT9 and *Salmonella* Mbandaka (5.0 × 10^8^ CFU of each serovar) suspended in Luria–Bertani (LB) broth (Oxoid, Australia). Serial tenfold dilutions of the inoculum were prepared and plated onto nutrient agar to confirm the total number of bacteria. Control birds received a sham inoculum containing only sterile LB broth. Clinical signs of infection were recorded throughout the experiment. At 30 weeks of age, [16 weeks post-infection (pi)] all birds were euthanized with Lethabarb (Virbac, Australia). Bone marrow, spleen, liver and caecal tonsils were collected from each bird for bacteriological examination.

### Enumeration of *Salmonella* in fecal samples

A total of 320 fecal samples from individual hens were collected aseptically using sterile plastic bags on day 1 post-infection (pi) followed by 1, 2, 4, 6, 8, 10, 12, 14 and 16 weeks pi. *Salmonella* enumeration using the three tube most probably number (MPN) method was performed on all faecal samples as described previously [24]. *Salmonella* suspected samples were streaked onto xylose lysine deoxycholate (XLD) agar plates (Oxoid, Australia) and *Salmonella* Brilliance agar plates (Oxoid, Australia) for confirmation of *Salmonella* spp.

### Bacterial DNA extractions from fecal samples, egg shell wash and internal organs

DNA was extracted from fecal samples using the Isolate Fecal DNA Kit (Bioline, Australia) following manufacturer instruction. DNA extraction from eggshell washes (enriched in RVS broth) collected from both infection groups was performed using Chelex^®^ (Bio-Rad, Sydney, NSW, Australia) [25] The Wizard genomic DNA purification kit (Promega, Australia) was used to extract DNA from the tissue samples as per manufacturer instructions.

### Standard curve and qPCR for fecal samples for *Salmonella* Typhimurium and *Salmonella* Mbandaka

The PCR detection of *Salmonella* was performed using the Quantifast^®^ SYBER^®^ Green qPCR kit (Qiagen, Australia) in a total reaction volume of 10 µL containing 2 µL sample (5 ng/µL), 5 µL of 2× Quantifast SYBER Green Master Mix and 1 µM of reverse and forward primers. *Salmonella* Typhimurium serovar specific primers *TSR*3 were used to detect *Salmonella* Typhimurium DT9. Further, to differentiate *Salmonella* Mbandaka from *Salmonella* Typhimurium DT9 in the co-infection group, primers for class 1 integron were used to specifically detect *Salmonella* Mbandaka [26]. The qPCR conditions were 5 min of denaturation at 95 °C, followed by 40 cycles of denaturation at 95 °C for 10 s and 60 °C for 30 s each. Rotor-gene 1.7.75 (Corbett Research, Qiagen, Australia) software version was used for the data analysis. A standard curve was generated to establish the limit of detection and quantification of positive samples, by determining a serial tenfold dilution of spiked fecal samples with known concentrations of *Salmonella* Typhimurium or *Salmonella* Typhimurium + *Salmonella* Mbandaka.

### Fecal corticosterone analysis

Fecal samples collected at day 1 (pi) followed by 1, 2, 4, 6, 8, 10, 12, 14 and 16 weeks pi were thawed, mixed, and dried at 103 °C overnight. After cooling to room temperature, samples were ground to a fine powder. Corticosterone metabolites were extracted using methods recommended by the DetectX Corticosterone EIA kit manufacturer (Arbor Assays, Ann Arbor, USA). The concentration of fecal corticosterone metabolites (FCM) was measured by DetectX Corticosterone EIA kit as per manufacturer instruction.

### Survey of egg shell and egg internal contents for *Salmonella* contamination

Eggs laid daily during 6, 8, 10, 12 and 14 weeks pi were collected and processed for *Salmonella* detection from both the eggshell and internal contents (Total eggs: 892; Control = 118, *Salmonella* Typhimurium only = 365, *Salmonella* Typhimurium + *Salmonella* Mbandaka co-infection = 409) using previously described methods [16]. Eggshell wash enriched in Rappaport–*Vassiliadis* broth (RVS; Oxoid, Australia) was stored in 80% glycerol at −80 °C to differentiate between *Salmonella* Typhimurium DT9 and *Salmonella* Mbandaka by standard PCR.

### PCR for egg shell wash and internal organ samples for *Salmonella* Typhimurium and *Salmonella* Mbandaka

*Salmonella* positive eggshell wash and internal organ samples from both infection groups were screened for the amplification of *invA* and *TSR*3 gene for detection of *Salmonella* Typhimurium by multiplex PCR [26]. *TSR*3 gene was not amplified in *Salmonella* Mbandaka isolates [26]. Samples from both groups were also tested for the presence of *Salmonella* Mbandaka.

### Bacteriology of internal organs

Bone marrow, spleen, liver and caecal tonsils were collected at week 16 pi and processed for bacteriology. Briefly, 0.1–0.2 grams of tissue sample were homogenised and serial tenfold dilutions were prepared in phosphate buffer saline (PBS). One hundred micro litre of each dilution was spread onto XLD agar plates and incubated overnight at 37 °C. After 24 h, the bacterial colonies were enumerated and the number of *Salmonella* in tissues was expressed as mean log_10_ CFU/g of tissue.

### Serum and egg yolk sample collection and serologic examination by ELISA

On day 0 and at 1, 2, 4, 6, 8, 10, 12, and 14 weeks pi, 2 mL blood samples were collected from each bird and placed into serum clot activator tubes (Vacuette^®^ tube, Greiner Bio-One, Australia). A total of 145 (Control; *n* = 20, *Salmonella* Typhimurium only; *n* = 57, Co-infection group; *n* = 68) egg samples collected at weeks 6, 8, 10, 12 and 14 pi were processed for the antibody extraction from the yolk samples. Egg yolk antibodies were extracted as described previously [27]. Dilutions of chloroform-extract egg yolk antibody were prepared from the pools of known positive and known negative eggs from control birds. Samples were tested in duplicate for the following dilutions; 1:10, 1:50, 1:100, 1:500 and 1:1000. From the curve produced, the linear part was expanded. Readings of known positive and negative samples individually at the selected dilution produced a cut-off value for the test. Threshold value were determined by plotting sensitivity and specificity against the cut off value using two graph receiver operating characteristics (TG-ROC) analysis as described [28]. A dilution factor of 1:100 was selected because it was on the linear part of the standard curve.

Antibody detection from both serum and egg yolk samples was tested using the Chicken *Salmonella* Typhimurium Antibody Kit LPS Group B (BioChek, Holland) and antibody titres were calculated according to manufacturer instruction.

### Statistical analysis

The data for average log_10_ CFU/qPCR, corticosterone level, and serum and egg yolk was analyzed using a two way analysis of variance (ANOVA) followed by a Tukey’s multiple comparison of the mean. Significance between bacterial titres in organs was tested using a Mann–Whitney test. The correlation between MPN/g fecal count and *Salmonella* positive eggshell wash, average log_10_ CFU/qPCR and corticosterone concentration was determined by Pearson correlation test (r^2^ value). All data was analysed using either by GraphPad Prism version 6 software or IBM^®^SPSS Statistics^®^ version 21. *p* values <0.05 were considered statistically significant. A D’Agostino-Pearson omnibus normality test was conducted for all data. Serum and egg yolk antibody titres were normally distributed. MPN data were not normally distributed. MPN data was analysed by a Kruskal–Wallis with a Dunn’s comparison of the means.

## Results

### Shedding and viable bacterial counts of *Salmonella* in fecal samples

**Figure 1:**
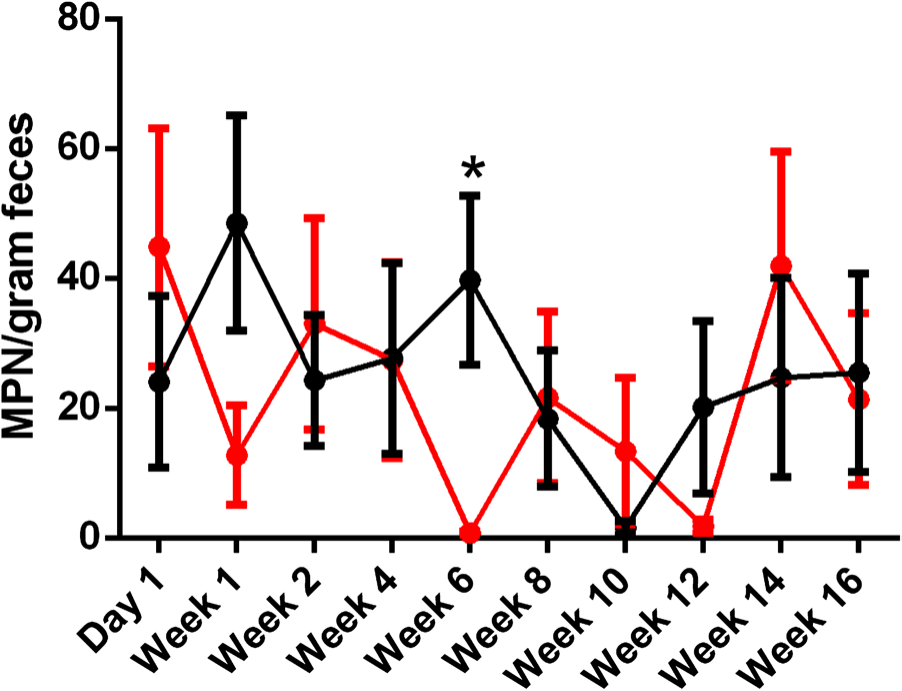
**Enumeration of** ***Salmonella*** **in feces using most probable number (MPN) method.** Fecal samples were collected from birds orally infected with 1 × 10^9^ CFU of *Salmonella* Typhimurium (Black line) or a combination of both *Salmonella* Typhimurium + *Salmonella* Mbandaka (5 × 10^8^ CFU of each serovar) (Red line) on day 1 and weeks 1, 2, 4, 6, 8, 10, 12, 14 and 16 pi. Data is presented as mean MPN/gram feces ± standard error of the mean. Bacterial shedding in both infection groups varied significantly over the course of the experiment (*p* < 0.01). At week 6 pi, bacterial shedding was significantly higher in birds infected with only *Salmonella* Typhimurium group (*p < 0.05).

Bacterial shedding varied significantly over time (*p* < 0.01) in both experimental treatment groups (Figure 1). The greatest number of viable bacteria observed in birds infected with only *Salmonella* Typhimurium occurred during week 1 pi, with a mean MPN/g of 48.53 ± 16.55. Samples collected from the *Salmonella* Typhimurium infection group in week 10 exhibited the lowest mean MPN/g, 1.535 ± 1.05. For birds infected with both *Salmonella* Typhimurium and *Salmonella* Mbandaka, the greatest number of viable *Salmonella* was detected on day 1 pi with a mean MPN/g of 44.80 ± 18.30. The lowest mean MPN/g, 0.78 ± 0.27, was observed in the multi-serovar infection group at week 6 pi.

Over the entire experiment, no significant effect of time or treatment was detected between single and multi-serovar treatment groups (*p* > 0.05). At week 6 pi, however, birds infected with only *Salmonella* Typhimurium exhibited a significantly greater mean MPN/g than birds infected with both *Salmonella* Typhimurium and *Salmonella* Mbandaka (*p* < 0.05). This difference correlated with the onset of lay. No *Salmonella* was detected in uninfected birds over the course of the experiment.

### Quantification of *Salmonella* in fecal samples using a serovar specific qPCR

A quantitative PCR was developed to detect total *Salmonella* Typhimurium in single infection fecal samples and differentiate between *Salmonella* Typhimurium and *Salmonella* Mbandaka co-infection samples. A standard curve was generated by spiking uninfected, control feces spiked with known quantities of *Salmonella* Typhimurium. A cut-off Ct of 32 was used to exclude the detection of false positives and corresponded to 100 CFU of *Salmonella*. For fecal samples spiked with both *Salmonella* Typhimurium and *Salmonella* Mbandaka, a cut-off Ct of 33 was used to exclude the detection of false positives. A Ct of 33 represented 1000 CFU of *Salmonella.* Data are presented as mean log_10_ CFU/gram feces ± standard error of the mean.

**Figure 2:**
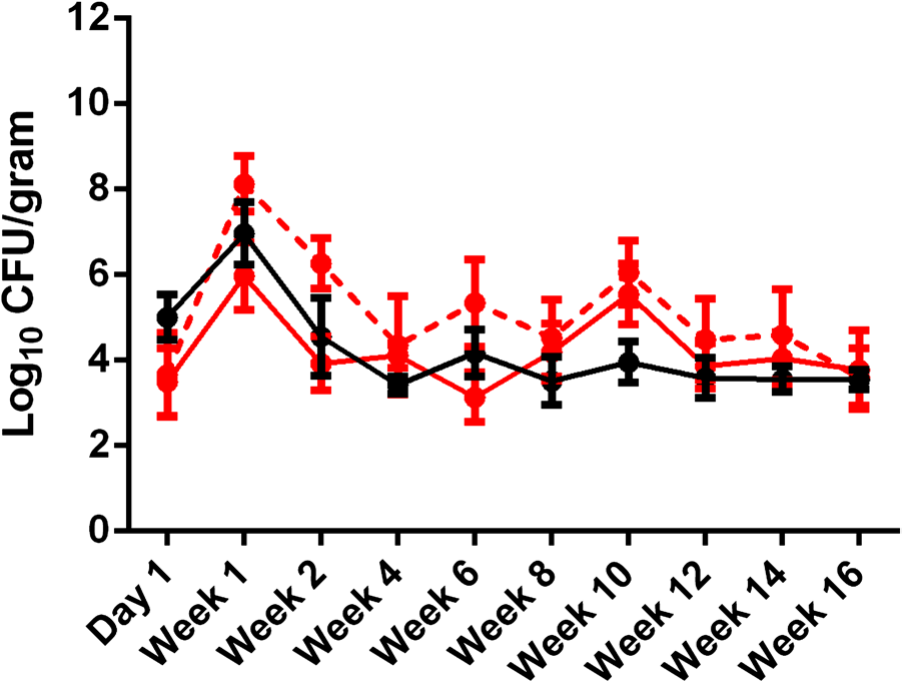
**Quantification and differentiation of** ***Salmonella*** **by qPCR.** Bacterial loads of fecal samples collected from birds infected with *Salmonella* Typhimurium only (black line) or co-infected with *Salmonella* Typhimurium (red line) and *Salmonella* Mbandaka (red hashed line) were quantified using a serovar specific qPCR. *Salmonella* Typhimurium was detected using a primers designed to the *TSR3* gene while *Salmonella* Mbandaka was detected using the *dhrfV* gene. Data are presented as mean log_10_ CFU/gram feces ± SEM. The amount of bacteria varied significantly over time (*p* < 0.01).

The number of *Salmonella* detected by qPCR varied significantly in both treatment groups over the course of the experiment (Figure 2). The greatest amount of *Salmonella* detected in all groups was observed at week 1 pi (Figure 2) with *Salmonella* Mbandaka in the co-infection group exhibiting the highest mean log_10_ CFU/gram feces (8.13 ± 0.65). Interestingly, *Salmonella* Mbandaka had the highest mean log_10_ CFU/gram feces between weeks 1 through 14 pi, though this difference was not significant than *Salmonella* Typhimurium. After week 1, *Salmonella* detection was relatively stable and consistent and did not vary significantly. No significant correlation was observed between MPN counts and qPCR results.

### Fecal corticosterone metabolites in dried fecal extracts

Measuring fecal corticosterone metabolites (FCM) is a non-invasive method enabling the measurement of one stress parameter [29, 30]. It has been previously shown that during point of lay, birds experience increased physiological stress and are thought to be immunocompromised [31]. Infection, however, has also been shown to affect plasma corticosterone levels [12]. Therefore, it was hypothesized that corticosterone should increase in all chickens around the onset of lay, and infection may lead to further increase in level of corticosterone.

**Figure 3:**
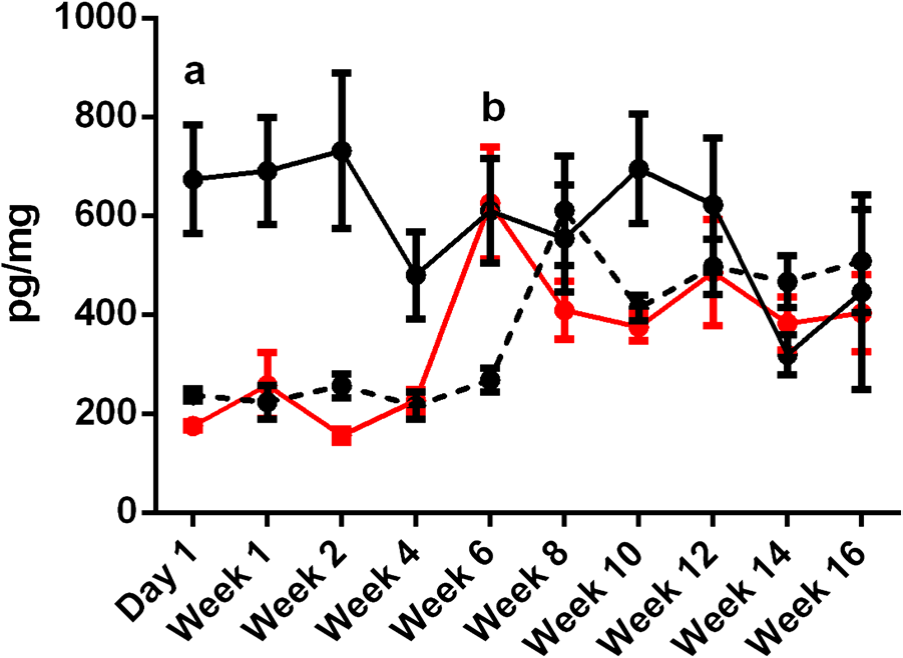
**Measurement of fecal corticosterone metabolites (FCM).** FCM concentrations were measured at day 1 and weeks 1, 2, 4, 6, 8, 10, 12, 14, and 16 pi. Data is presented as mean pg/mg feces ± SEM. A significant effect of time (*p* < 0.05) and treatment (*p* < 0.001) was detected for FCM concentrations. At day 1 pi, the mean FCM in birds infected with *Salmonella* Typhimurium (black line) was significantly higher than either control (black hashed line) or co-infected birds (red line) (a; *p* < 0.001). At week 6 pi, the mean FCM in co-infected birds increased. At this time point, both infection groups were exhibited significantly higher FCM concentrations than control birds (b; *p* < 0.01).

Fecal samples collected for enumeration of bacteria were also processed for FCM. A significant effect of time (*p* < 0.05) and treatment (*p* < 0.001) were observed between FCM concentrations (Figure 3). At day 1 pi, the FCM in birds infected with *Salmonella* Typhimurium (674.2 ± 109.3 pg/mg) was significantly higher than the FCM observed for control birds (238.0 ± 12.62 pg/mg) or birds infected with a mixed inoculum of both *Salmonella* Typhimurium and *Salmonella* Mbandaka (175.4 ± 8.58 pg/mg) (*p* < 0.001).

At week 6 pi, the mean FCM (625.2 ± 113.2 pg/mg) increased in birds co-infected with both *Salmonella* Typhimurium and *Salmonella* Mbandaka. At this time point, no significant difference between the two infection groups was detected. The mean FCM in control birds (268.7 ± 24.19 pg/mg), however, was significantly less than both treatment groups (*p* < 0.01). At weeks 8, 12, 14 and 16 pi, the mean FCM obtained for all groups varied but did not differ significantly (Figure 3).

No significant correlation was detected between the mean FCM concentration and MPN counts in singly or co-infected birds (r^2^ = − 0.036, *p* = 0.699). A significant but weak positive correlation (r^2^ = 0.26, *p* = 0.02) was observed between the mean log copy number/gram and FCM concentration in birds infected with *Salmonella* Typhimurium only.

### Detection of *Salmonella* from eggshell wash and internal contents

Eggs were collected at weeks 6, 8, 10, 12, and 14 pi and tested for the presence of *Salmonella* on the shell surface and within the internal contents. *Salmonella* was isolated throughout the experiment from the eggshell wash of experimentally infected hens. In birds infected with only *Salmonella* Typhimurium, the percentage of eggshell contamination ranged from 9.52 to 21.74%. Birds infected with both *Salmonella* Typhimurium and *Salmonella* Mbandaka exhibited a similar level of eggshell contamination, 10.89–33.33% (Table 1). By culture methods, the percentage of eggshell contamination was highest in both the groups at week 6 pi (onset of lay). No significant difference in eggshell contamination frequency was detected between *Salmonella* infection treatment groups. PCR results of egg shell samples indicated that the recovery rate of *Salmonella* Typhimurium (11.74%) was higher than *Salmonella* Mbandaka (6.60%) in co-infection group (Table 1).

**Table 1:** **Percentage of isolation of** ***Salmonella*** **and** ***Salmonella*** **Typhimurium by culture and PCR method respectively from eggshell samples of orally infected birds at different weeks of pi**

Weeks pi	*Salmonella* Typhimurium only group		Co-infection group		
	*Salmonella* detection by culture method	*Salmonella* Typhimurium detection by PCR	*Salmonella* detection by culture method	*Salmonella* Typhimurium detection by PCR	*Salmonella* Mbandaka detection by PCR
Week 6	21.74^c^ (5/23)^a^	17.39 (4/23)	33.33 (8/24)	8.33 (2/24)^b^	8.33 (2/24)^b^
Week 8	9.52 (8/84)	8.33 (7/84)	10.89 (11/101)	8.91 (9/101)	0.00 (0/101)
Week 10	15.85 (13/82)	15.85 (13/82)	15.22 (14/92)	10.87 (10/92)	5.43 (5/92)
Week 12	13.48 (12/89)	12.36 (11/89)	22.92 (22/96)	14.58 (14/96)	13.54 (13/96)
Week 14	11.49 (10/87)	10.34 (9/87)	21.88 (21/96)	13.54 (13/96)	7.29 (7/96)
Total	13.15 (48/365)	12.05 (44/365)	13.20 (54/409)	11.74 (48/409)	6.60 (27/409)

^a^Number of positive eggs/total number of eggs tested.

^b^Results confirmed by PCR.

^c^Values in  %.

No linear correlation was observed between the *Salmonella* MPN count in feces and eggshell contamination of infected birds (r^2^ = 0.001, *p* = 0.99). *Salmonella* was not detected in egg internal contents of either infection treatment group at any point during this experiment. Eggshells and internal contents from control hens were also negative for *Salmonella*.

### *Salmonella* Typhimurium antibody titres in serum and egg yolk samples

**Figure 4:**
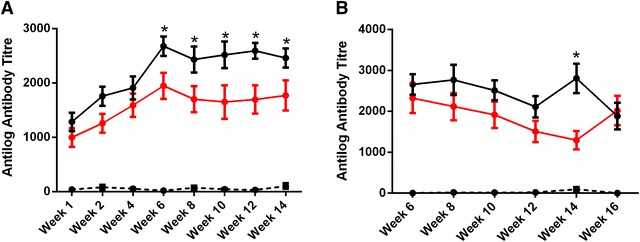
**Quantification of** ***Salmonella*** **Typhimurium specific antibodies in serum and yolk.** *Salmonella* Typhimurium specific antibody titers (antilog antibody titres ± SEM) were characterised over the course of the experiment in both serum (**A**) and yolk (**B**) in control birds (black hashed line) as well as hens infected with only *Salmonella* Typhimurium (black line) or a combination of both *Salmonella* Typhimurium and *Salmonella* Mbandaka (red line). In the serum, the amount of *Salmonella* Typhimurium antibody was significantly higher in singly infected birds compare with co-infected birds from week 6 pi till the end of the experiment (*p < 0.01). Mean antibody titres detected in yolk samples collected from *Salmonella* Typhimurium infected birds were only significantly different from co-infected birds at week 14 pi (*p < 0.01).

The titres of *Salmonella* Typhimurium specific serum and yolk antibodies were measured over the course of the experiment (Figures 4A and B). The lowest mean antibody titre (antilog) in birds infected with only *Salmonella* Typhimurium was observed at week 1 pi (1286 ± 168.1) and peaked at week 6 pi (2678 ± 179.5). After week 6 pi, antibody titres remained constant during the remainder of the experiment. A similar pattern was observed for *Salmonella* Typhimurium antibodies measured from the co-infection group. In birds infected with both *Salmonella* Typhimurium and *Salmonella* Mbandaka, the mean titre was lowest at week 1 pi (997.7 ± 170.5) and highest at week 6 pi (1949 ± 239.1). Mean antibody titres of birds infected with *Salmonella* Typhimurium only were significantly higher than those obtained for the co-infection group at weeks 6, 8, 10, 12, and 14 pi (*p* < 0.01). Control birds were negative for *Salmonella* Typhimurium antibodies over the course of the experiment.

Eggs collected from both infection groups tested positive for *Salmonella* yolk antibodies (Figure 4B). A significant effect of treatment was detected between the experimental groups (*p* ≤ 0.01).

### Persistence of *Salmonella* in internal organs

**Figure 5:**
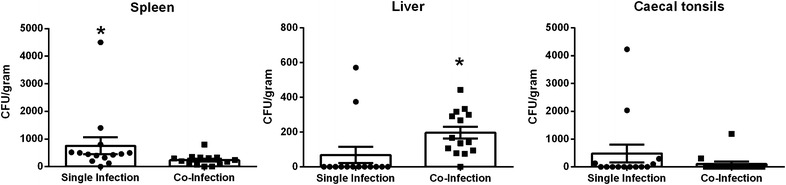
**Bacterial persistence in spleen, liver and caecal tonsils.** The total amount of viable bacteria was quantified from internal organs at week 16 pi. Data is presented as mean CFU/gram tissue ± standard error of the mean. Individual data points have also been included to highlight the variation within each group. In splenic samples, bacterial loads in birds infected with only *Salmonella* Typhimurium were significantly higher than the titre observed in the co-infection group (*p < 0.01). Only 2/14 liver samples from the *Salmonella* Typhimurium treatment group were positive for bacteria, while 13/14 positive samples were detected for co-infected birds. The mean for the liver was significantly higher in the co-infection group (*p < 0.01). No significant difference was detected in bacterial loads from the caecal tonsils.

At 30 weeks of age (week 16 pi), the experiment was terminated and birds were euthanized. Spleen, liver, bone marrow and caecal tonsils from all hens were collected and processed for *Salmonella* to characterise the persistence of the bacteria in these organs. All samples collected from control hens were negative for *Salmonella*. Bacteria were detected in all tissues except for the bone marrow samples. The total number of positive samples was greatest in the spleen, followed by the liver and caecal tonsils (Figure 5). The mean splenic bacterial load observed in birds infected with only *Salmonella* Typhimurium (757.4 ± 301.1 CFU/g tissue) was significantly greater than the mean titre observed for birds inoculated with both *Salmonella* Typhimurium and *Salmonella* Mbandaka (236.0 ± 54.51 CFU/g tissue) (*p* < 0.01).

Birds infected with both *Salmonella* Typhimurium and *Salmonella* Mbandaka exhibited the highest number of individuals positive for *Salmonella* in the liver (92.9%) (Figure 5). The mean bacterial titre for the co-infection group was 197.1 ± 34.17 CFU/g tissue and was significantly higher than the mean titre observed for birds infected with *Salmonella* Typhimurium, 68.46 ± 46.97 CFU/g tissue (Mann–Whitney, *p* < 0.01). The lowest overall level of *Salmonella* colonisation was observed in the caecal tonsils with 35.7% positive individuals in the single infection group and 14.3% positive birds in the multi-serovar group (Figure 5). Bacterial titres in caecal tonsils collected from birds infected with only *Salmonella* Typhimurium ranged from 0 to 4.2 × 10^3^ CFU/g tissue with a mean titre of 485.7 ± 321.9 CFU/g tissue. Birds infected with both *Salmonella* Typhimurium and *Salmonella* Mbandaka ranged from 0 to 1.2 × 10^3^ CFU/g tissue with a mean of 107.1 ± 85.79 CFU/g tissue. No significant difference was detected between the two infection treatment groups.

The culture positive internal organs were further tested by PCR to differentiate *Salmonella* Typhimurium and *Salmonella* Mbandaka in the co-infection group (Table 2). In splenic samples, 1 of 14 was positive for *Salmonella* Typhimurium while 4/14 samples were positive for *Salmonella* Mbandaka. Three of 14 liver samples from the co-infection group were positive for *Salmonella* Mbandaka, however, no *Salmonella* Typhimurium was detected. In the caecal tonsils, 1/14 samples were positive for *Salmonella* Typhimurium and 1/14 tested positive for *Salmonella* Mbandaka.

**Table 2:** **Recovery and enumeration of** ***Salmonella*** **from internal organs**

Organ	*Salmonella* Typhimurium only group			Co-infection group			
	*Salmonella* detection by culture method	Mean log_10_ CFU/g and SEM	*Salmonella* Typhimurium detection by PCR	*Salmonella* detection by culture method	Mean log_10_ CFU/g and SEM	*Salmonella* Typhimurium detection by PCR	*Salmonella* Mbandaka detection by PCR
Spleen	13/14 (92.85%)	2.52 ± 0.22 (*n* = 13)	3/14 (21.43%)	12/14 (85.71%)	2.01 ± 0.24 (*n* = 12)	1/14 (7.14%)	4/14 (28.57%)
Liver	2/14 (14.29%)	0.38 ± 0.26 (*n* = 2)	2/14 (14.29%)	13/14 (92.85%)	2.10 ± 0.17 (*n* = 13)	0/14 (0.00%)	3/14 (21.43%)
Caecal tonsils	5/14 (35.71%)	0.97 ± 0.38 (*n* = 5)	2/14 (14.29%)	2/14 (14.29%)	0.40 ± 0.27 (*n* = 2)	1/14 (7.14%)	1/14 (7.14%)

## Discussion

This study indicated that layers infected with *Salmonella* Typhimurium DT9 became persistently infected causing intermittent bacterial shedding in faeces. At week 6 pi, the MPN count in *Salmonella* Typhimurium infected group was significantly higher than multi-serovar infection group. Week 6 corresponded to the onset of lay in experimental birds and it was postulated that this increase could be related to physiological stress induced by onset of lay [16].

FCM levels in the *Salmonella* Typhimurium group were higher than either the control or multi-serovar treatment groups from day 1 until week 6 pi. Bacterial lipopolysaccharide (LPS) can induce inflammation within a host and has been associated with increased serum and corticosterone [12]. The LPS of *Salmonella enterica* is variable, serovar dependent and contributes to different degrees of virulence. This may account for lower mean FCM concentrations in the multi-serovar group. Increase in FCM in all treatment groups between 6-8 weeks could be attributed to the onset of lay, and infection may lead to further increase in level of FCM. However, it important to note that no positive correlation was observed between bacterial shedding and FCM levels in this study.

During this study, higher rates of eggshell contamination at the onset of lay could be attributed to increased *Salmonella* shedding in feces at that point [5, 16]. There was no linear correlation between *Salmonella* shedding in feces and egg shell contamination of infected birds and this is in agreement with earlier reports [32]. Of note, in this study *Salmonella* was not detected from egg internal contents.

The *Salmonella* Typhimurium IgG antibody titres increased after week 1 pi and peaked at week 6 pi. Birds were seropositive till the end of the trial at week 14 pi but the immune response did not result in complete clearance of *Salmonella spp*. It is also important to note that the antibody response contributes to the clearance of extracellular bacteria, intracellular bacteria can persist in the host thus cell mediated immune response is essential for clearance of *Salmonella* Typhimurium (reviewed in [33]). Overall decreased IgG antibody response in multi-serovar infection group could perhaps be due to the competitive and immunoprotective mechanism between both *Salmonella* strains. However, the absence of an infection treatment with only *Salmonella* Mbandaka in this study limits this conclusion.

In the multi-serovar group, a low MPN was obtained at week 6 yet qPCR results revealed similar loads of both *Salmonella* Typhimurium and *Salmonella* Mbandaka. The discrepancy may be due to the detection of both live and dead bacteria using PCR method. However it is unclear why that has happened specifically at week 6 pi. Laying hens mounted immune response to invasive strain of *Salmonella* Typhimurium DT9 without inducing clinical signs. Variation in qPCR and MPN results could be attributed to the sensitivity of the tests used. Several factors such as heterogeneous distribution of the pathogen in sample, number of stressed cells, sample matrix, enrichment time and enrichment media can influence the accuracy of quantitation [34].

In *Salmonella* Typhimurium infected group there were increased levels of FCM concentrations, antibody titres and bacterial shedding (as detected by MPN method) at week 6 pi (onset of lay) which supports the theory that the presence of stress hormones can stimulate *Salmonella* growth and enhance bacterial colonisation in the intestine [35]. However present data suggests that this theory may not apply when host is infected with multiple *Salmonella* serovars. Concentration of corticosterone levels in sera can increase or decrease the antibody response [36]. In this study the high corticosterone levels did not suppress the humoral immune response against *Salmonella* Typhimurium.

Stress can stimulate the recrudescence of bacteria from internal organs resulting in high bacterial load in feces [37]. Our findings indicated that *Salmonella* Typhimurium persisted in internal organs despite high levels of circulating specific IgG antibody. Previous studies reported *Salmonella* Typhimurium clearance from liver and other internal organs due to Th-1 dominated responses and high levels of interferon-γ expression at around 14–28 days pi [38]. Some studies reported low frequency of *Salmonella* Enteritidis in liver and other internal organs for up to 22 weeks pi [39]. It has also been suggested that age at exposure did not affect recovery of *Salmonella* Typhimurium from liver [40]. Our observations could not be compared with previous reports because such studies were performed using broilers for short period of time. It could be hypothesised that persistence of *Salmonella* Typhimurium in internal organs including liver could be due to the timing of challenge (prior to lay in this case). Sexual maturity can induce immunosuppression by altering cellular and humoral immune response [33]. This could ultimately cause bacteria to avoid clearance and dominate host leading to a recrudescence of infection. However further studies are required to confirm this hypothesis. It is interesting to note that in mixed infection group, only *Salmonella* Mbandaka was detected from liver.

Previous literature stated that intestinal persistence of *Salmonella* Typhimurium in chickens was longer when birds were challenged at day old compared to day 7 and that older birds are considerably more resistant to salmonellae than are young chicks [41, 42]. Our study indicated that infection of adult birds (14 weeks old in this study) can also result in continued harbouring of the *Salmonella* Typhimurium and intermittent faecal shedding. This shedding can be associated with the stress event such as onset of lay. However interplay between stress, immune response and *Salmonella* Typhimurium shedding in single or mixed infection group at the onset of lay is more complex to understand.

To conclude, *Salmonella* Typhimurium DT9 persistently infected hens causing intermittent bacterial shedding in faeces. At the onset of lay shedding of *Salmonella* Typhimurium was affected in mixed infection group. Increased immune response did not result in clearance of *Salmonella spp* (except for *Salmonella* Typhimurium at week 6 pi). There was no correlation between FCM and *Salmonella* shedding. This long term *Salmonella* Typhimurium infection model provided useful insights on the continued persistence and or recrudescence of *Salmonella* Typhimurium, although further investigation is necessary to understand the immunobiology of long term and systemic *Salmonella* Typhimurium infection.

## References

[1] Moffatt CR, Musto J, Pingault N, Miller M, Stafford R, Gregory J, Polkinghorne BG, Kirk MD (2016). *Salmonella* Typhimurium and outbreaks of egg-associated disease in Australia, 2001 to 2011. Foodborne Pathog Dis.

[2] Threlfall E, Wain J, Peters T, Lane C, De Pinna E, Little C, Wales A, Davies R (2014). Egg-borne infections of humans with salmonella: not only an *S.* Enteritidis problem. World Poultr Sci J.

[3] Chousalkar KK, Sexton M, McWhorter A, Hewson K, Martin G, Shadbolt C, Goldsmith P (2015). *Salmonella* Typhimurium in the Australian egg industry: multidisciplinary approach to addressing the public health challenge and future directions. Crit Rev Food Sci Nutr.

[4] Williams A, Davies A, Wilson J, Marsh P, Leach S, Humphrey T (1998). Contamination of the contents of intact eggs by *Salmonella* Typhimurium DT104. Vet Rec.

[5] Okamura M, Sonobe M, Obara S, Kubo T, Nagai T, Noguchi M, Takehara K, Nakamura M (2010). Potential egg contamination by *Salmonella* enterica serovar Typhimurium definitive type 104 following experimental infection of pullets at the onset of lay. Poult Sci.

[6] Okamura M, Miyamoto T, Kamijima Y, Tani H, Sasai K, Baba E (2001). Differences in abilities to colonize reproductive organs and to contaminate eggs in intravaginally inoculated hens and in vitro adherences to vaginal explants between *Salmonella* Enteritidis and other *Salmonella* serovars. Avian Dis.

[7] Leach SA, Williams A, Davies AC, Wilson J, Marsh PD, Humphrey TJ (1999). Aerosol route enhances the contamination of intact eggs and muscle of experimentally infected laying hens by *Salmonella* Typhimurium DT104. FEMS Microbiol Lett.

[8] Borsoi A, Quinteiro-Filho WM, Calefi AS, Piantino Ferreira AJ, Astolfi-Ferreira CS, Florio JC, Palermo-Neto J (2015). Effects of cold stress and *Salmonella* Heidelberg infection on bacterial load and immunity of chickens. Avian Pathol.

[9] Quinteiro-Filho WM, Rodrigues M, Ribeiro A, Ferraz-de-Paula V, Pinheiro M, Sa L, Ferreira A, Palermo-Neto J (2012). Acute heat stress impairs performance parameters and induces mild intestinal enteritis in broiler chickens: role of acute hypothalamic-pituitary-adrenal axis activation. J Anim Sci.

[10] Holt PS (1993). Effect of induced molting on the susceptibility of White Leghorn hens to a *Salmonella* Enteritidis infection. Avian Dis.

[11] Nakamura M, Nagamine N, Takahashi T, Suzuki S, Kijima M, Tamura Y, Sato S (1994). Horizontal transmission of *Salmonella* Enteritidis and effect of stress on shedding in laying hens. Avian Dis.

[12] Shini S, Kaiser P, Shini A, Bryden WL (2008). Biological response of chickens (*Gallus gallus domesticus*) induced by corticosterone and a bacterial endotoxin. Comp Biochem Physiol B: Biochem Mol Biol.

[13] Mashaly M, Hendricks G, Kalama M, Gehad A, Abbas A, Patterson P (2004). Effect of heat stress on production parameters and immune responses of commercial laying hens. Poult Sci.

[14] Quinteiro-Filho W, Gomes A, Pinheiro M, Ribeiro A, Ferraz-de-Paula V, Astolfi-Ferreira C, Ferreira A, Palermo-Neto J (2012). Heat stress impairs performance and induces intestinal inflammation in broiler chickens infected with *Salmonella* Enteritidis. Avian Pathol.

[15] Gole VC, Torok V, Sexton M, Caraguel CG, Chousalkar KK (2014). Association between indoor environmental contamination by *Salmonella* enterica and contamination of eggs on layer farms. J Clin Microbiol.

[16] Gole VC, Caraguel CG, Sexton M, Fowler C, Chousalkar KK (2014). Shedding of *Salmonella* in single age caged commercial layer flock at an early stage of lay. Int J Food Microbiol.

[17] Pulido-Landínez M, Sanchez-Ingunza R, Guard J, Nascimento V (2013). Assignment of serotype to *Salmonella* enterica isolates obtained from poultry and their environment in Southern Brazil. Lett Appl Microbiol.

[18] Chousalkar K, Gole V, Caraguel C, Rault JL (2016). Chasing *Salmonella* Typhimurium in free range egg production system. Vet Microbiol.

[19] Glass K, Fearnley E, Hocking H, Raupach J, Veitch M, Ford L, Kirk MD (2016). Bayesian source attribution of salmonellosis in South Australia. Risk Anal.

[20] Doyle EM (2013) White paper on human illness caused by *Salmonella* from all food and non-food vectors. Update 2013 in: FRI Food Safety Reviews (Ed), Madison, pp 1-45

[21] Guard J, Sanchez-Ingunza R, Shah DH, Rothrock MJ, Gast RK, Jones DR (2015). Recovery of *Salmonella* enterica serovar Enteritidis from hens initially infected with serovar Kentucky. Food Chem.

[22] Pulido-Landínez M, Sanchez-Ingunza R, Guard J, do Nascimento VP (2013). Presence of *Salmonella* Enteritidis and *Salmonella* Gallinarum in commercial laying hens diagnosed with fowl typhoid disease in Colombia. Avian Dis.

[23] Pande VV, Devon RL, Sharma P, McWhorter AR, Chousalkar KK (2016). Study of *Salmonella* Typhimurium infection in laying hens. Front Microbiol.

[24] USDA, U.S. Department of agriculture, food safety and inspection services (2008) most probable number procedure and tables. In: Microbiology Laboratory Guidebook. https://www.fsis.usda.gov/wps/wcm/connect/8872ec11-d6a3-4fcf-86df-4d87e57780f5/MLG-Appendix-2.pdf?MOD=AJPERES. Accessed 14 Jan 2015

[25] Pande VV, Gole VC, McWhorter AR, Abraham S, Chousalkar KK (2015). Antimicrobial resistance of non-typhoidal *Salmonella* isolates from egg layer flocks and egg shells. Int J Food Microbiol.

[26] Akiba M, Kusumoto M, Iwata T (2011). Rapid identification of *Salmonella* enterica serovars, Typhimurium, Choleraesuis, Infantis, Hadar, Enteritidis, Dublin and Gallinarum, by multiplex PCR. J Microbiol Methods.

[27] Gole V, Chousalkar K, Roberts J (2012). Prevalence of antibodies to *Mycoplasma synoviae* in laying hens and possible effects on egg shell quality. Prev Vet Med.

[28] Greiner M, Sohr D, Gobel P (1995). A modified ROC analysis for the selection of cut-off values and the definition of intermediate results of serodiagnostic tests. J Immunol Methods.

[29] Dehnhard M, Schreer A, Krone O, Jewgenow K, Krause M, Grossmann R (2003). Measurement of plasma corticosterone and fecal glucocorticoid metabolites in the chicken (*Gallus domesticus*), the great cormorant (*Phalacrocorax carbo*), and the goshawk (*Accipiter gentilis*). Gen Comp Endocrinol.

[30] Rettenbacher S, Mostl E, Hackl R, Ghareeb K, Palme R (2004). Measurement of corticosterone metabolites in chicken droppings. Br Poult Sci.

[31] Shini S, Huff G, Shini A, Kaiser P (2010). Understanding stress-induced immunosuppression: exploration of cytokine and chemokine gene profiles in chicken peripheral leukocytes. Poult Sci.

[32] Gast RK, Guard-Bouldin J, Holt PS (2005). The relationship between the duration of fecal shedding and the production of contaminated eggs by laying hens infected with strains of *Salmonella* Enteritidis and *Salmonella* Heidelberg. Avian Dis.

[33] Chappell L, Kaiser P, Barrow P, Jones MA, Johnston C, Wigley P (2009). The immunobiology of avian systemic salmonellosis. Vet Immunol Immunopathol.

[34] Malorny B, Lofstrom C, Wagner M, Kramer N, Hoorfar J (2008). Enumeration of *Salmonella* bacteria in food and feed samples by real-time PCR for quantitative microbial risk assessment. Appl Environ Microbiol.

[35] Verbrugghe E, Dhaenens M, Leyman B, Boyen F, Shearer N, Van Parys A, Haesendonck R, Bert W, Favoreel H, Deforce D, Thompson A, Haesebrouck F, Pasmans F (2016). Host stress drives *Salmonella* recrudescence. Sci Rep.

[36] Campos-Rodriguez R, Kormanovski A, Stephano AQ, Abarca-Rojano E, Berczi I, Ventura-Juarez J, Drago-Serrano ME, Kumar Yashwant (2012). The central nervous system modulates the immune response to Salmonella. Salmonella—a diversified superbug.

[37] Rostagno M, Wesley I, Trampel D, Hurd H (2006). *Salmonella* prevalence in market-age turkeys on-farm and at slaughter. Poult Sci.

[38] Beal RK, Powers C, Wigley P, Barrow PA, Smith AL (2004). Temporal dynamics of the cellular, humoral and cytokine responses in chickens during primary and secondary infection with *Salmonella* enterica serovar Typhimurium. Avian Pathol.

[39] Gast RK, Beard CW (1990). Isolation of *Salmonella* enteritidis from internal organs of experimentally infected hens. Avian Dis.

[40] Gast RK, Beard CW (1989). Age-related changes in the persistence and pathogenicity of *Salmonella* Typhimurium in chicks. Poult Sci.

[41] Groves PJ, Sharpe SM, Muir WI, Pavic A, Cox JM (2016). Live and inactivated vaccine regimens against caecal *Salmonella* Typhimurium colonisation in laying hens. Aust Vet J.

[42] Gast RK (2008) Paratyphoid infections. In: Saif Y (Ed) Diseases of Poultry, 12th edn. Blackwell, Ames, pp 636–655

